# ZnuA and zinc homeostasis in *Pseudomonas aeruginosa*

**DOI:** 10.1038/srep13139

**Published:** 2015-08-20

**Authors:** Victoria G. Pederick, Bart A. Eijkelkamp, Stephanie L. Begg, Miranda P. Ween, Lauren J. McAllister, James C. Paton, Christopher A. McDevitt

**Affiliations:** 1 Research Centre for Infectious Diseases, School of Biological Sciences, University of Adelaide, Adelaide, South Australia, Australia

## Abstract

*Pseudomonas aeruginosa* is a ubiquitous environmental bacterium and a clinically significant opportunistic human pathogen. Central to the ability of *P. aeruginosa* to colonise both environmental and host niches is the acquisition of zinc. Here we show that *P. aeruginosa* PAO1 acquires zinc via an ATP-binding cassette (ABC) permease in which ZnuA is the high affinity, zinc-specific binding protein. Zinc uptake in Gram-negative organisms predominantly occurs via an ABC permease, and consistent with this expectation a *P. aeruginosa* Δ*znuA* mutant strain showed an ~60% reduction in cellular zinc accumulation, while other metal ions were essentially unaffected. Despite the major reduction in zinc accumulation, minimal phenotypic differences were observed between the wild-type and Δ*znuA* mutant strains. However, the effect of zinc limitation on the transcriptome of *P. aeruginosa* PAO1 revealed significant changes in gene expression that enable adaptation to low-zinc conditions. Genes significantly up-regulated included non-zinc-requiring paralogs of zinc-dependent proteins and a number of novel import pathways associated with zinc acquisition. Collectively, this study provides new insight into the acquisition of zinc by *P. aeruginosa* PAO1, revealing a hitherto unrecognized complexity in zinc homeostasis that enables the bacterium to survive under zinc limitation.

Zinc is the second most abundant first-row transition metal in biological organisms^1^. Approximately 6% of prokaryotic proteins are predicted to bind zinc^2^ and this can be attributed to the ability of the metal ion to serve in both structural and catalytic roles^1^,^3^. Although zinc lacks redox activity, due to its completely filled *d*-shell, it can still mediate significant toxicity in biological systems by inappropriately binding to the metal binding sites of proteins or DNA, thereby perturbing or inhibiting their function^4^,^5^. Consequently, efficient management and regulation of zinc homeostasis is a critical aspect of prokaryotic chemical biology.

Zinc, which occurs as the divalent cation Zn^2+^ in biological systems, is present at widely varying concentrations in the environment. The bioavailability of Zn^2+^ is dictated by a number of prevailing variables and, in soils and plants, Zn^2+^ content is highly dependent on both geological and meteorological contributions, typically occurring within a range between 15 and 200 mg Zn^2+^ per kg (dry weight)^6^,^7^. Significant variation in metal ion abundance also occurs in the context of host-pathogen interactions. Mammalian hosts, such as humans, employ nutritional immunity as a component of their innate defence, wherein they restrict the bioavailability of certain transition metal ions, by using chelating proteins, such as calprotectin and psoriasin, to hamper bacterial colonisation during the initial stages of infection^8^,^9^. At later stages of infection, transition metal ion fluxes, notably Zn^2+^ and copper, have been associated with the prosecution of metal-toxicity towards bacterial pathogens^9^. As both a ubiquitous environmental organism and a clinically significant opportunistic human pathogen, *P. aeruginosa* encounters widely varying levels of Zn^2+^ abundance depending on its niche. To date, there has been limited information regarding how the bacterium manages its cellular Zn^2+^ content in response to fluctuations in extracellular Zn^2+^ abundance.

Specific, high-affinity acquisition of Zn^2+^ was first demonstrated in *E. coli* and shown to occur via the ATP-binding cassette (ABC) permease, ZnuABC^10^. The Znu permease comprises the solute-binding protein (SBP) ZnuA, and an ABC transporter, which consists of ZnuB (the transmembrane protein) and ZnuC (the nucleotide-binding domain), in a ZnuAB_2_C_2_ organisation^10^. The SBP was shown to be Zn^2+^-specific and responsible for delivery of Zn^2+^ ions to the ZnuBC transporter, located in the cytoplasmic membrane. The Znu permease and its homologs are the most common Zn^2+^ uptake pathway in prokaryotes^11^,^12^,^13^,^14^. Loss of the Znu permease in many species, including *E. coli*, *Salmonella* Typhimurium and *Yersinia pestis*, typically results in a pronounced growth defect^12^,^13^. Zinc acquisition is controlled by metalloregulatory proteins, such as the Zn^2+^-uptake regulator (Zur), which is a Zn^2+^-specific regulatory protein, belonging to the ferric uptake regulator (Fur) family of transcriptional regulators^15^. In *P. aeruginosa* Zur (formerly Np20 and PA5499), was recently shown to be a Zn^2+^-responsive metalloregulatory protein that mediated the Zn^2+^-dependent repression of a putative *znuABC* permease^16^. Hence, in *P. aeruginosa*, as in many prokaryotes, Zn^2+^ appears to negatively regulate its own accumulation via transcriptional control over the Zn^2+^-import pathway genes.

Complementary to the Zur-dependent regulation of Zn^2+^ uptake, prokaryotic organisms also efflux Zn^2+^ ions to prevent Zn^2+^ overload. Efflux of cellular Zn^2+^ from prokaryotes can occur via a number of distinct transporters depending on the organism, and include resistance-nodulation division pumps (e.g. CzcCBA), cation diffusion facilitator transporters (e.g. ZitB and CzcD), and P-type ATPase transporters (e.g. ZntA)^17^,^18^,^19^,^20^. Expression of each of these efflux systems is tightly controlled by a cognate transcriptional regulator^21^,^22^,^23^. Collectively, studies of the Zn^2+^-uptake and -efflux pathways and their associated regulators have revealed that prokaryotic Zn^2+^ homeostasis involves acutely tight regulation and management of intracellular Zn^2+^ ions. Although the absolute intracellular accumulation of Zn^2+^ varies, depending on the organism^24^,^25^, studies of *E. coli* have indicated that the concentration of ‘free’ or labile Zn^2+^ present in the cytoplasm was extraordinarily low (in femtomolar range).

Bioinformatics analyses of the *P. aeruginosa* PAO1 genome identified three genes homologous to *E. coli znuABC*: PA5498 (*znuA*), PA5500 (*znuB*), and PA5501 (*znuC*), which have also been shown to be under the control of the Zur transcriptional regulator^16^,^26^. The putative *znuA* gene is present in a separate operon to the putative ABC transporter components (*znuB* and *znuC*), while *zur* is encoded immediately upstream of *znuB* and *znuC*, with the three genes forming an operon^16^. PA5502, which encodes a putative lipoprotein, is present following *znuC*, but this gene is transcribed independently of *zur* and *znuBC*, suggesting that it does not play a role in Zn^2+^ acquisition. Although *P. aeruginosa* mutant strains lacking *znuA*, *znuB,* or *znuC* were recently shown to have a modest reduction in the final biomass, when grown overnight in rich media treated with the divalent cation-chelating agent ethylenediaminetetraacetic acid (EDTA)^16^, direct evidence for their involvement in Zn^2+^-specific acquisition has been lacking. Hence, although studies of Zn^2+^ regulation in *P. aeruginosa* have implicated an ABC uptake system in Zn^2+^ uptake, its precise molecular role has not yet been elucidated^16^. Here, we report on the cellular accumulation of Zn^2+^ in *P. aeruginosa* PAO1, which represents ~10% of the total cellular transition metal content, the primary mechanisms of Zn^2+^ acquisition, and the impact of Zn^2+^ limitation upon transcriptional regulation and cellular physiology.

## Results and Discussion

### *P. aeruginosa* encodes a Zn^2+^-specific ABC permease

**Figure 1:**
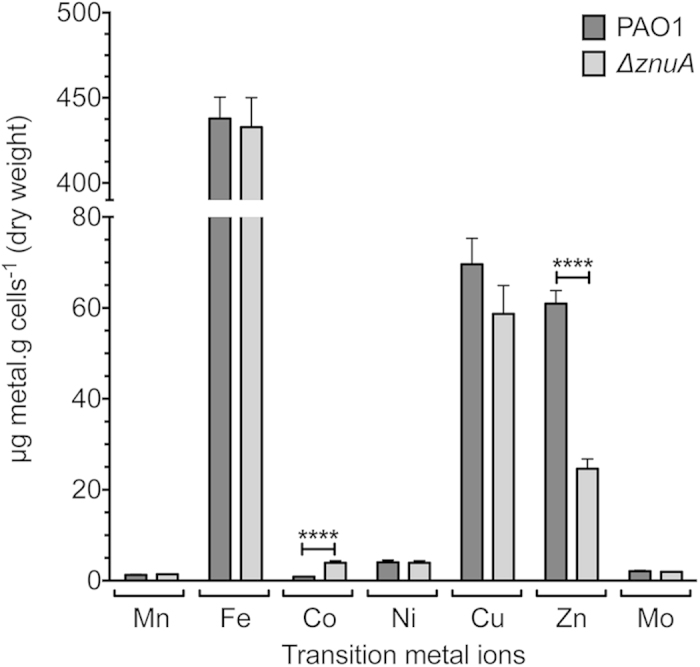
Whole cell metal accumulation analyses. *In vitro* accumulation of metals by wild-type PAO1 (dark grey) and Δ*znuA* cultures (light grey) were assessed via growth in CDM. Metal content was expressed as μg of metal per gram of dry cells, as determined by ICP-MS. Data are the mean ± s.e.m., with duplicate readings taken from each biological replicate grown on three separate days. Statistical significance was determined using the two-tailed unpaired Student’s *t*-test, where ****represents *P* < 0.0001.

**Figure 2:**
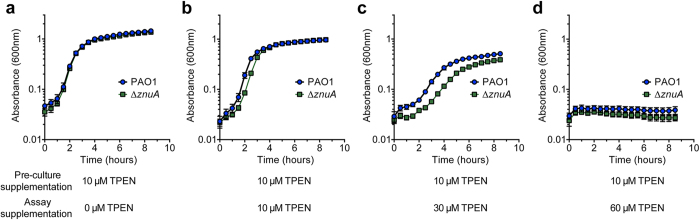
The effect of Zn^2+^ limitation on *P. aeruginosa* growth. The growth phenotypes of *P. aeruginosa* PAO1 (blue) and the Δ*znuA* strain (green) were compared by absorbance at 600 nm. (**a**) CDM overnight seed cultures were supplemented with 10 μM TPEN, prior to growth analyses in unsupplemented CDM. (**b**) CDM overnight seed cultures were supplemented with 10 μM TPEN, prior to growth analyses in CDM supplemented with 10 μM TPEN. **(c)** CDM overnight seed cultures were supplemented with 10 μM TPEN, prior to growth analyses in CDM supplemented with 30 μM TPEN. (**d**) CDM overnight seed cultures were supplemented with 10 μM TPEN, prior to growth analyses in CDM supplemented with 60 μM TPEN. Cultures were grown as indicated and, in all experiments, data are the mean ± s.e.m., with n ≥ 3.

To directly assess the role of the *P. aeruginosa* PAO1 putative ZnuA protein in Zn^2+^ acquisition, we constructed a mutant strain lacking *znuA* (Δ*znuA*). Whole cell metal accumulation of wild-type *P. aeruginosa* and the Δ*znuA* strain was assessed in Chelex-100 treated, chemically-defined media (CDM) by inductively coupled plasma-mass spectrometry (ICP-MS). Metal accumulation analyses revealed a significant 59.6% decrease in cellular Zn^2+^ due to loss of the SBP (*P* < 0.0001; Fig. 1). Disruption of the Znu permease had no impact on the cellular accumulation of other transition metal ions apart from cobalt, which increased in cellular abundance (*P* < 0.0001; Fig. 1), suggesting that the *P. aeruginosa* Zn^2+^ regulatory and/or homeostatic mechanisms may also associated with cobalt homeostasis. Collectively, these data indicate that the *P. aeruginosa znuA* gene, and by extension the Znu permease, is associated with acquisition of Zn^2+^, while loss of *znuA* results in a significant disruption of cellular Zn^2+^ homeostasis. Due to the widespread utilization of zinc in cellular processes, it was anticipated that impairment of Zn^2+^ accumulation would result in perturbation of growth, as has been observed in other bacteria^12^,^13^. However, despite the highly restricted Zn^2+^ content (800 nM) of the CDM, the Δ*znuA* mutant strain did not exhibit a growth defect (Supplementary Fig. S1 online). Supplementation of the CDM with 10 μM of the preferential Zn^2+^ chelating agent N,N,N′,N′-tetrakis(2-pyridylmethyl)ethylenediamine (TPEN) in the pre-culture also failed to elicit a significant phenotypic impact on growth, despite being present at 10-fold in excess of the Zn^2+^ present in the media (Fig. 2a). Subsequent growth of the pre-treated PAO1 and Δ*znuA* mutant strains in the presence of 10 μM TPEN resulted in a slight growth perturbation of the mutant strain (Fig. 2b), an effect enhanced at 30 μM TPEN (Fig. 2c). Growth of both strains was inhibited in the presence of 60 μM TPEN (Fig. 2d). Therefore, as the growth defects were elicited in both the wild-type and mutant strain (Fig. 2c,d, and Supplementary Fig. S1 online) principally at higher TPEN concentrations, i.e. 30 μM and 60 μM relative to Zn^2+^, it cannot be excluded that the chelation of other essential transition row metal ions also contributed to these more pronounced phenotypic impacts. These observations would be consistent with the recent study of Ellison *et al.* (2013), wherein a slight growth perturbation was observed for a *znuA* mutant grown in undefined media with the broad acting divalent-chelating agent EDTA. In our study, the minor growth perturbation observed for the Δ*znuA* strain, relative to the wild-type, under Zn^2+^ limitation suggests that one or more high-affinity Zn^2+^ acquisition pathways may exist in *P. aeruginosa* that permit acquisition of Zn^2+^ ions, present at nanomolar concentrations, from the extracellular environment.

### ZnuA is a high affinity Cluster A-I Zn^2+^-binding protein

**Figure 3:**
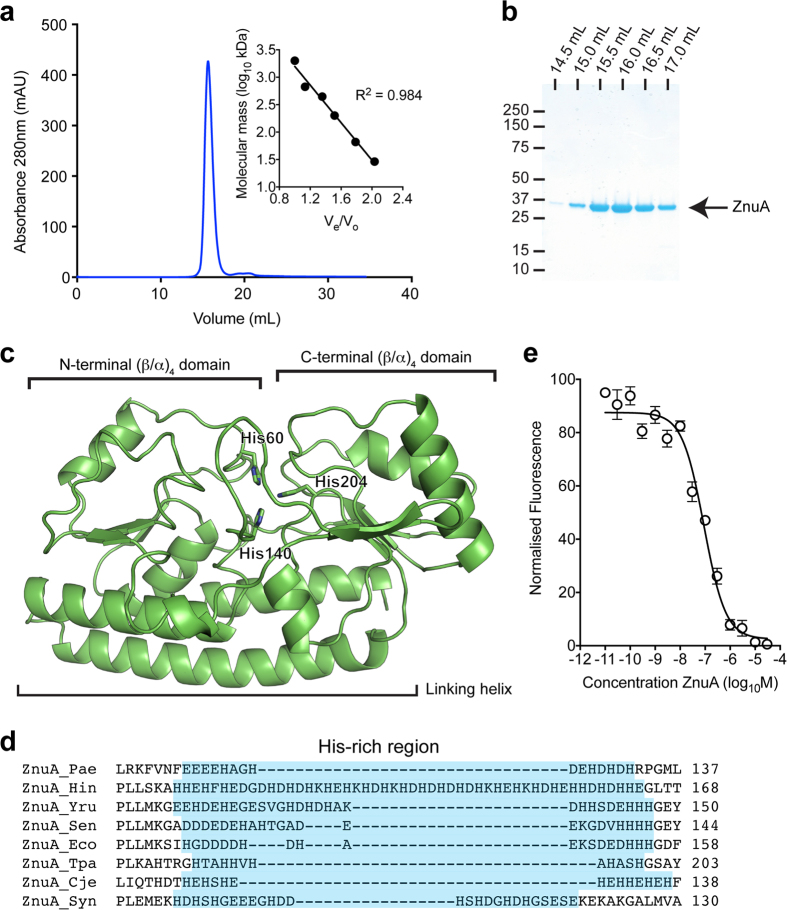
Bioinformatics and biochemical characterisation of ZnuA. (**a**) Absorbance (280 nm) trace of ZnuA analysed by gel permeation chromatography. Inset represents linear regression of soluble molecular mass standards used to determine the apparent molecular mass of ZnuA. ZnuA was determined to be monomeric with a molecular mass of 34.5 kDa. (**b**) Coomassie-stained SDS-PAGE analysis of monomeric ZnuA (indicated by arrow) by comparison with soluble molecular mass standards. Fractions analysed were 0.5 mL, with elution volume at start of each fraction indicated above the lane. (**c**) Cartoon representation of the homology-based model of *P. aeruginosa* ZnuA showing a typical cluster A-I fold and the predicted Zn^2+^ binding residues. (**d**) Primary sequence alignment of *P. aeruginosa* ZnuA, and ZnuA orthologs from other bacterial species with the His-rich region indicated in blue. ZnuA proteins from: Pae, *P. aeruginosa* (GI:15600691); Hin, *H. influenza* (GI:491963406); Yru, *Y. ruckeri* (GI:490857750); Sen, *S. enterica* (GI:541470409); Eco, *E. coli* (GI:635897169); Tpa, *T. palladium* (GI:504108253); Cje, *C. jejuni* (GI:504330062); Syn, *Synechocystis 6803* (GI:499174152). (**e**) Competitive binding experiment with apo-ZnuA using Mag-fura-2-Zn^2+^. The normalised fluorescence emission (520 nm) of Mag-fura-2 was monitored in response to the addition of increasing concentrations of apo-ZnuA. Data are the mean (±s.e.m.) for three independent experiments.

To ascertain whether *P. aeruginosa* ZnuA is a high-affinity Zn^2+^-SBP, biochemical and biophysical characterisation was undertaken. Recombinant C-terminal dodecahistidine-tagged ZnuA was expressed without the putative Sec-type signal peptide and purified by immobilised metal affinity chromatography and gel permeation chromatography (GPC) (Fig. 3a,b). GPC indicated that recombinant ZnuA was isolated as a single monodisperse species with a relative molecular mass of 34.5 kDa, which matched closely with the predicted molecular mass (34.4 kDa) of monomeric dodecahistidine-tagged ZnuA. The dodecahistidine tag was cleaved from ZnuA prior to subsequent characterisation. Endogenous metals were removed by denaturation at pH 4.0 in the presence of 30 mM EDTA, prior to refolding by dialysis in 50 mM Tris-HCl, pH 7.2, 100 mM NaCl. ICP-MS analysis of refolded tag-cleaved ZnuA found that it was metal-free (apo), containing less than 0.01 mol of metal ions per mol of protein. A thermostabilisation assay was employed to assess cation interaction with ZnuA (Table 1). Zinc induced the largest increase in ZnuA stability, consistent with the role of ZnuA in Zn^2+^ acquisition as indicated by whole cell ICP-MS. Intriguingly, cobalt induced the next largest increase in ZnuA thermostability. However, as cobalt accumulation in *P. aeruginosa* is an order of magnitude less than Zn^2+^, and increased rather than decreased in the Δ*znuA* strain, ZnuA does not appear to have a physiological role in cobalt uptake.

Primary sequence analysis of *P. aeruginosa* PAO1 ZnuA indicates that it belongs to the Cluster A-I (formerly cluster IX) subgroup of SBPs associated with ABC transporters (Supplementary Fig. S2 online)^27^. High-resolution structural analyses have shown that cluster A-I SBPs have a bi-lobed architecture, with the N- and C-terminal (β/α)_4_-domains linked by a long alpha-helix and the protein surface bisected by the cleft between the two lobes. In Zn^2+^-specific cluster A-I SBPs, Zn^2+^ is generally bound by three Nε2 atoms, contributed by conserved histidine residues, and an oxygen atom from a coordinating carboxylate residue or a water molecule within this cleft^28^,^29^,^30^. An energy-minimised homology model of ZnuA was generated based on a high-resolution crystal structure of ZnuA from *E. coli* (PDB 2OGW) (Fig. 3c). Primary sequence alignment and the structural prediction indicated that the high-affinity Zn^2+^-binding site, located in the interdomain cleft of *P. aeruginosa* ZnuA, would comprise His60, His140, and His204 (Nε2 contributing residues). The metal ion coordination modality observed in the *E. coli* ZnuA homolog (Glu77, His78, His161, and His225) is unlikely to occur in *P. aeruginosa* ZnuA due to the absence of an oxygen-contributing residue at the position proximal to the first His residue (His60) or elsewhere in the vicinity of the Zn^2+^ ion binding site^29^. Instead, the coordinating oxygen-ligand would mostly likely be a water molecule, as observed in the *Synechocystis* 6803 ZnuA homolog^30^. Similar to the high-resolution structures of other cluster A-I SBPs, the metal-binding site of ZnuA would be buried ~10–15 Å beneath the molecular surface of the protein^5^,^29^,^30^,^31^. In addition to the metal-coordinating residues, a disordered region of 15 acidic residues, which is similar to the His-rich region (or loop) of other Zn^2+^-specific SBPs, was also identified in the primary sequence of *P. aeruginosa* ZnuA (Fig. 3d). The length of this region has been observed to vary in Zn^2+^-specific SBPs from 12 residues (*T. pallidum*)^32^ to 50 residues (*H. influenzae*)^33^, but this region was not present in the homology model due to its absence from all high-resolution crystal structures.

Zinc-specific SBPs from Gram-negative organisms contain a single high affinity Zn^2+^-binding site. In addition, the His-rich loop has been reported to bind Zn^2+^, but with much lower affinity (~3–4 orders of magnitude lower). Here, ZnuA was analysed using a competitive Zn^2+^-binding assay with the Zn^2+^-responsive fluorophore Mag-Fura-2. A titration with increasing concentrations of ZnuA revealed a *K*_D_ for Zn^2+^ of 22.6 ± 6.4 nM (Fig. 3e). This is consistent with the nanomolar affinity of other ZnuA homologs for Zn^2+^^34^,^35^,^36^. We then investigated the stoichiometry of Zn^2+^ binding by ZnuA. ICP-MS analysis of a ZnuA-Zn^2+^ equilibrium binding experiment showed that ZnuA bound 1.6 ± 0.1 mol Zn^2+^.mol protein^−1^. The stoichiometry indicated the presence of an additional Zn^2+^-binding site, consistent with observations from other Zn^2+^-specific SBPs from Gram-negative organisms, e.g. *E. coli* ZnuA (~1.85 mol Zn^2+^.mol protein^−1^)^34^ and *H. influenzae* Pzp1 (1.6–1.9 mol Zn^2+^.mol protein^−1^)^33^, which is likely due to the presence of the low-affinity (micromolar) His-rich Zn^2+^-binding region. It has been suggested that the role of the His-rich region is to aid in delivery of Zn^2+^ to the primary binding site of the SBP or in facilitating Zn^2+^ transfer to ZnuB 30. However, due to its highly disordered structure, conclusive evidence has remained elusive. Irrespective, the His-rich loop has a much lower (micromolar) affinity, with its precise role in ZnuA only poorly defined^35^. Indeed, the His-rich loop is not essential for the function of the high affinity Zn^2+^-binding site, although recent studies have indicated that this region may play a role in promoting Zn^2+^ interaction with ZnuA in order to aid in Zn^2+^ binding at the high-affinity site *in vivo*^37^. Taken together, these data show that *P. aeruginosa* ZnuA is a high-affinity Zn^2+^-specific cluster A-I SBP. Similar to other Gram-negative SBPs, *P. aeruginosa* ZnuA is competent for binding multiple Zn^2+^ atoms. Collectively, these analyses indicate that the Znu permease is a major Zn^2+^ acquisition pathway of *P. aeruginosa*.

### Zinc depletion results in transcriptional modulation

**Figure 4:**
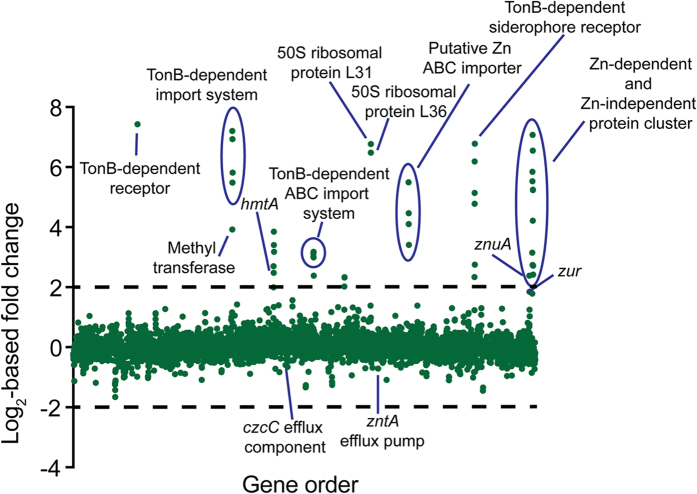
Differential expression of genes in response to the Zn^2+^ depletion of the Δ*znuA* strain. RNA sequencing of *P. aeruginosa* PAO1 and the isogenic Δ*znuA* deletion strain was used to determine relative gene expression (expressed as log_2_-fold change). Each green dot represents a gene, with each gene distributed on the x-axis in accordance with locus tag numbering for PAO1. Genes more highly expressed in the Δ*znuA* strain are present above the x-axis, with those below the x-axis expressed at a lower level in the Δ*znuA* strain. Genes of interest are annotated with their putative or characterised functions.

Bioinformatic studies have predicted Zn^2+^ to be utilised by approximately 6% of prokaryotic proteins^2^. Consequently, it was anticipated that the Zn^2+^ deficiency of the Δ*znuA* strain would be accompanied by a significant transcriptional response. To identify the pathways affected by Zn^2+^ depletion, the transcriptomes of wild-type *P. aeruginosa* PAO1 and the Δ*znuA* strain were analysed by RNA sequencing (Table 2 and Fig. 4). Overall 88 of the 5697 genes were up-regulated ≥2-fold, with 44 up-regulated ≥4-fold. Surprisingly, only 22 genes were down-regulated ≥2-fold in the Δ*znuA* strain. Quantitative reverse transcription-PCR analysis of several representative genes validated the RNA sequencing results (Supplementary Fig. S3 online).

**Figure 5:**
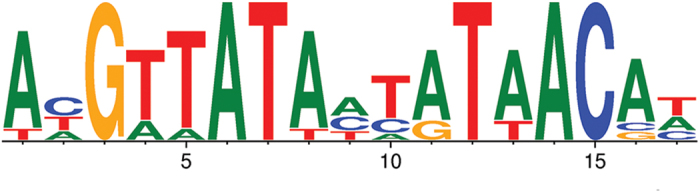
The *P. aeruginosa* PAO1 Zur motif. The size of the nucleotide (T in red, A in green, C in blue and G in yellow) indicates its conservation across the 9 Zur binding site sequences listed in Supplementary Table S1 online. The 17 bp motif shows a palindrome with a central non-conserved nucleotide in position 9. The *P. aeruginosa* PAO1 Zur motif was created using WebLogo 3.0^66^.

In order to examine the role of the *P. aeruginosa* Zur in modulating the transcriptional response to Zn^2+^ limitation, we examined the genome for the presence of putative Zur binding sites. Recently, the *P. protegens* Pf-5 Zur motif was determined^38^, providing a template from which a *P. aeruginosa* PAO1 optimized Zur binding motif could be generated. The *P. protegens* Pf-5 Zur motif was subjected to iterative refinement by only selecting putative sites in the *P. aeruginosa* PAO1 genome that were positioned intergenically, up-regulated ≥2-fold as determined by our RNA-sequencing data, and possessing an *E*-value ≤ 0.002, until no new candidate sites were identified. On the basis of these parameters, a PAO1 Zur motif was generated from 9 Zur binding sites (Fig. 5 and Supplementary Table S1 online). Subsequent examination of the transcriptomic responses of *P. aeruginosa* to Zn^2+^ deficiency showed that Zur is the primary regulator of Zn^2+^ homeostasis in this bacterium, as all but 9 of the transcriptionally responsive genes up-regulated ≥4-fold possessed a Zur binding site (Table 2).

### Zinc homeostatic mechanisms

Although deletion of *znuA* reduced cellular Zn^2+^ abundance, the Δ*znuA* strain was capable of acquiring sufficient Zn^2+^ to facilitate a growth phenotype similar to that of the wild-type strain. Hence, given the restriction of Zn^2+^ abundance in the CDM to nanomolar concentrations, it is likely that *P. aeruginosa* PAO1 possesses one or more additional high affinity Zn^2+^ acquisition mechanisms to ensure the cellular Zn^2+^ requirement is met. Analysis of the RNA-sequencing data allowed identification of three putative transport systems, in addition to the ZnuABC permease, that may facilitate translocation of Zn^2+^ across the inner membrane into the cell: PA2911-PA2914, *hmtA* and PA4063-PA4066. Each of these putative transport systems was identified as being under the transcriptional control of Zur and was significantly up-regulated in the Δ*znuA* strain (Supplementary Fig. S4 online).

Primary sequence analyses predicted that PA2911-PA2914 encodes an iron ABC permease (PA2912-PA2914) that is co-transcribed with a putative TonB-dependent receptor (PA2911). However, studies of iron limitation in *P. protegens* indicated that the homologous cluster was not associated with iron recruitment^39^. Furthermore, the presence of a Zur site in the regulatory elements of the PA2911-PA2914 cluster (*E*-value = 0.00027; Table 2) is consistent with the observed transcriptional response to Zn^2+^ depletion. However, the mechanism by which the PA2911-PA2914 cluster may acquire Zn^2+^ is not immediately apparent, as primary sequence analysis of the putative PA2913 SBP component indicates that it does not belong to the cluster A-I subgroup of ABC permease cation-recruiting SBPs. Instead, PA2913 more closely resembles a cluster A-II SBP, suggesting that it may interact with a chelated form of Zn^2+^ (Supplementary Fig. S2 online). Although we have no direct evidence for a chelated-Zn^2+^ complex in *P. aeruginosa*, recently a Zn^2+^-chelating molecule known as yersiniabactin, was characterized in *Yersinia pestis*^40^. Yersiniabactin Zn^2+^ uptake was shown to be dependent upon the major facilitator family transporter, YbtX. Although a Zn^2+^-chelate ABC-dependent uptake system has not yet been identified, it is not inconceivable that PA2911, which shares homology with a TonB-dependent receptor, may function in concert with PA2912-PA2914 to facilitate transport of chelated Zn^2+^ from the extracellular environment to the cytoplasm. Of interest, PA2914 also shares homology with the transmembrane domain protein of the Vitamin B12 (cobalamin) ABC permease. Hence, the up-regulation of the PA2911-PA2914 system in response to Zn^2+^ depletion may enable the import of cobalt-containing cobalamin, possibly accounting for the increase in cellular cobalt levels observed in the Δ*znuA* strain (Fig. 1). Further studies of PA2911-PA2914 will be required to elucidate whether Zn^2+^ or cobalt could be acquired via this type of pathway.

A second putative ABC permease gene cluster (PA4063-PA4066) featuring a Zur site (*E*-value = 0.0011) was also up-regulated in response to Zn^2+^ limitation. By contrast with other ABC permeases, the individual putative SBP genes associated with this gene cluster, PA4063 and PA4066, are too small to form an SBP of sufficient size to stably interact with a ligand and the transmembrane domains of the ABC transporter. Furthermore, monomeric PA4066 has an insufficient number of histidine residues to coordinate Zn^2+^ ions, while PA4063 appears to have an abundance of histidine residues. Thus, it remains unclear how these proteins may contribute to Zn^2+^ homeostasis. Zinc-depletion was also associated with the up-regulation *hmtA*, an atypical P-type ATPase importer involved in Zn^2+^ and copper import (Supplementary Fig. S4 online)^41^. The *hmtA*-containing gene cluster (PA2434-PA2439) was also shown to feature a weak putative Zur binding site (*E*-value = 0.11). Collectively, these putative Zur-regulated transporters may aid in Zn^2+^ acquisition in the absence of the functional Znu permease, thereby minimizing the impact of Zn^2+^ depletion and the growth phenotype perturbation.

In addition to the transport systems identified in the inner membrane, four genes encoding putative TonB-dependent outer membrane proteins were found to be up-regulated in the Δ*znuA* strain (PA0781, PA1922, PA2911 and PA4837). The gene most highly up-regulated, as determined in our transcriptome study, was PA0781 (172-fold), which shares 27% identity with the TonB-dependent Zn^2+^-binding protein ZnuD from *Neisseria meningiditis*^42^. ZnuD facilitates Zn^2+^ recruitment to the periplasm under Zn^2+^-restricted conditions, thereby enabling subsequent import of Zn^2+^ to the cytoplasm^42^. PA2911 is associated with an ABC permease (PA2912-PA2914), discussed above, while the two remaining putative TonB-dependent receptors are also present within Zur-regulated gene clusters. The putative TonB-dependent receptor PA1922 is located within an operon that contains a *cobN*-like gene (PA1923), which encodes a putative cobaltochelatase involved in cobalamin biosynthesis. The up-regulation of this operon may account for the increase in cellular cobalt levels observed in the Δ*znuA* mutant (Fig. 1). Alternatively, Zn^2+^ may substitute for cobalt in PA1923^43^, although the precise role of this operon in metal ion homeostasis remains to be determined. The TonB-dependent receptor encoded by PA4837 is located in an operon with a putative nicotianamine synthase (PA4836). Although the function of nicotianamine in bacteria has not been explored, these secondary metabolites have previously been shown to be involved in Zn^2+^ homeostasis in plant and yeast cells^44^. It is tempting to speculate that the putative drug/metabolite exporter encoded by PA4834 is involved in the transport of nicotianamine to the periplasm of *P. aeruginosa*. However, the exact interaction of the TonB-dependent receptor encoded by PA4837 and nicotianamine remains unknown. Consequently, further studies are required to ascertain the role of these pathways and whether they contribute to Zn^2+^ and/or cobalt acquisition.

TonB-dependent outer membrane receptors rely on TonB, ExbB and ExbD to energize transport. *P. aeruginosa* PAO1 features two identified *exbB* and *exbD* genes (*exbB1* and *exbB2*, and *exbD1* and *exbD2*) and three *tonB* genes (*tonB1*, *tonB2* and *tonB3*), but these were not significantly up-regulated under Zn^2+^ restriction. However, PA1924 encoding a putative ExbD homolog was up-regulated by ~44-fold under Zn^2+^ deficiency. Co-transcribed with the putative TonB-dependent receptor PA1922, PA1924 may serve as a component of the TonB uptake pathway in *P. aeruginosa*.

Comparative analyses of the Zn^2+^ acquisition mechanisms described above revealed that, in general, these proteins are highly conserved within the species (data not shown). Major sequence variation was only observed within PA4063, specifically within the second of the two histidine rich regions of the protein, wherein the number of histidine residues varied between 3 and 10 across the species. Since PA4063 may play a role in delivery of Zn^2+^ to the ABC-transporter encoded by PA4064-PA4065, the substantial differences observed within the histidine rich region could have a profound impact on the efficiency of Zn^2+^ uptake via this system in different *P. aeruginosa* strains.

### Zinc limitation and transcriptional regulation of ribosomal proteins

Prokaryotic ribosomal proteins commonly occur in two forms, which either bind metals ions such as Zn^2+^ (C+ isoform), or lack the ability to interact with metal ions (C− isoform) due to the absence of the metal-binding residues^45^. It is the ability of the C− form to substitute for the Zn^2+^-dependent C+ form that enables ribosomal function to be maintained under Zn^2+^ limitation^46^. This has led to the suggestion that ribosomal proteins may act as a Zn^2+^ reservoir and allow Zn^2+^ redeployment during periods of Zn^2+^ depletion^46^. Similar to *P. protogens* Pf-5, *P. aeruginosa* harbours genes for both the C+ and C− paralogs of the 50s ribosomal proteins L36 and L31^38^,^45^. The C+ copies of L36 and L31 (*rpmJ*/PA4242 and *rpmE*/PA5049, respectively) each feature canonical Zn^2+^-binding resides (either His or Cys). The C− isoforms L36 and L31 (PA3600 and PA3601, respectively) are predicted to be co-transcribed under the control of an adjacent putative Zur site (*P* = 0.0013), and lack almost all of the Zn^2+^-binding residues. Consistent with these analyses the C− (Zn^2+^-independent) L36 (PA3600) and L31 (PA3601) isoforms were highly up-regulated (89.2- and 109-fold, respectively) under Zn^2+^-depleted conditions. This implicates redeployment of Zn^2+^ via the switch to C− ribosomal proteins as a potential strategy for managing Zn^2+^ depletion.

### Up-regulation of genes encoding Zn^2+^-independent paralogs and Zn^2+^-dependent proteins

The importance of Zn^2+^ as a structural and catalytic cofactor in a range of proteins necessitates an efficient strategy on behalf of the bacterium to adapt to Zn^2+^ limitation. This is presumed to involve a combination of substitution by Zn^2+^-independent paralogs and redeployment of Zn^2+^ to proteins that have an absolute requirement for Zn^2+^. We identified a Zur-regulated cluster of genes (PA5532-PA5541), which encodes a number of genes up-regulated in response to Zn^2+^ depletion. A similar, yet distinct cluster was recently identified in a study examining Zn^2+^ depletion in *P. protegens* Pf-5^38^. The Pf-5 cluster includes genes encoding an ABC import system (PFL_6178-PFL_6180) and two putative enzymes (PFL_6181 and PFL_6184). By contrast, the up-regulated genes of the *P. aeruginosa* PAO1 cluster include DksA2 (PA5536), the Zn^2+^-independent global transcriptional regulator that substitutes for the Zn^2+^-dependent DksA (PA4723) under Zn^2+^-limiting conditions^47^,^48^. DksA and DksA2 have major roles in regulating the starvation response of *P. aeruginosa*^47^,^48^. In addition, other up-regulated genes encoding Zn^2+^-independent paralogs include *pyrC2* (PA5541), a dihydroorotase involved in pyrimidine biosynthesis^49^, a putative γ-carbonic dehydratase (PA5540), responsible for the conversion of carbon dioxide to bicarbonate^50^ and *folE2* (PA5539). Up-regulated 93.6-fold and featuring a putative Zur binding site (*E*-value = 0.00017), *folE2* is a putative Zn^2+^-independent GTP cyclohydrolase. The Zn^2+^-dependent FolE catalyses the first step of the *de novo* tetrahydrofolate (THF) biosynthetic pathway as well as the production of modified ribonucleosides found in tRNA molecules^51^, with a similar role predicted for FolE2. In contrast, the PA5532-PA5541 cluster also includes AmiA (PA5538), an *N*-acetylmuramoyl-L-alanine amidase involved in membrane remodelling that has a strict requirement for Zn^2+^ 52. Two of the other genes up-regulated in this cluster, PA5532 and PA5535, belong to the COG0523 family and encode proteins with putative roles in cobalamin biosynthesis. However, approximately 30% of COG0523 family genes are predicted to be involved in Zn^2+^ homeostasis rather than cobalamin biosynthesis, while ~8% are directly Zur-regulated^43^. Although the precise role of these genes remains unclear, it is highly probable that they serve in facilitating cation homeostasis, particularly under Zn^2+^ restriction^43^.

### Down-regulation of genes in response to Zn^2+^ depletion

Intriguingly, only a small proportion of genes were down-regulated by ≥2-fold in response to Zn^2+^ depletion and the majority of these encode tRNAs (38%). The nitrite reductase cluster (*nirCFGHJL*) showed a ≥2-fold reduction in transcription, although as none of the proteins involved in nitrate reduction directly utilize Zn^2+^, the underlying basis for this is unclear. The Zn^2+^ efflux pathways were only minimally down-regulated in the Δ*znuA* strain, with PA2522 (*czcC*) down-regulated 1.3-fold, and the *E. coli zntA* homolog, PA3690, down-regulated 1.6-fold. This indicates very limited Zn^2+^ efflux was required by the wild-type PAO1 strain in the CDM media used, with intracellular Zn^2+^ concentrations attributable to high affinity uptake pathways.

## Conclusions

**Figure 6:**
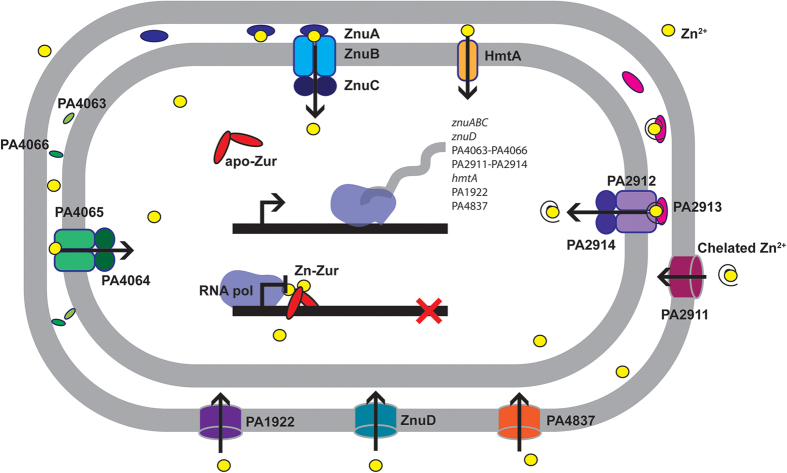
Proposed model of Zn^2+^ acquisition in *P. aeruginosa* PAO1. Schematic representation of the Zn^2+^ acquisition pathways of *P. aeruginosa* PAO1 based on transcriptomic and biochemical analyses. Under Zn^2+^-replete conditions, dimeric Zur, the primary Zn^2+^-responsive regulator, binds Zn^2+^, thereby repressing transcription of the Zn^2+^ import pathways. Zinc limitation facilitates the dissociation of Zn^2+^ from Zur, thereby permitting de-repression of the Zn^2+^ uptake pathway genes. Zinc entry into the periplasm occurs via four TonB-dependent outer membrane proteins: ZnuD (PA0781), PA2911, PA1922, and PA4837. Within the periplasm, Zn^2+^-specific SBPs (ZnuA, PA2913, PA4063 and PA4066) likely bind Zn^2+^, either as the free ion or chelated Zn^2+^, and deliver it to a cognate ABC import system (ZnuBC, PA2912/PA2914 and PA4064/PA4065), which facilitates vectorial transport to the cytosol. In addition, HmtA, a P-type ATPase is also able to import periplasmic Zn^2+^ ions into the cytoplasm.

In environments of changing Zn^2+^ abundance, efficient acquisition and efflux mechanisms are crucial for maintaining cellular Zn^2+^ homeostasis. Similar to other prokaryotes, the Znu permease is a high-affinity Zn^2+^ acquisition pathway in *P. aeruginosa* PAO1, and the biochemical and biophysical properties of ZnuA are consistent with this role. Although disruption of the Znu permease resulted in significant impairment in cellular Zn^2+^ accumulation, this was not observed to elicit a major perturbation of growth. The global impact of Zn^2+^ limitation on *P. aeruginosa* PAO1 was revealed by the role of Zur in the regulation of genes associated with cellular Zn^2+^ homeostasis. Zur binding sites were identified adjacent to 79.5% (35 of 44) of the genes observed to be up-regulated by more than 4-fold in response to Zn^2+^ depletion. However, not all genes differentially regulated in response to Zn^2+^ depletion were located downstream of putative Zur binding sites, suggesting other regulatory processes also contribute to management of cellular stress under conditions of Zn^2+^ depletion. Transcriptome analyses showed that under Zn^2+^ limitation, *P. aeruginosa* PAO1 up-regulated a number of previously unidentified putative metal ion import pathways while also inducing the expression of Zn^2+^-independent paralogs of Zn^2+^-dependent proteins, such as the ribosomal proteins L31 and L36, PyrC2 and DksA2. In parallel, genes encoding proteins that have been reported to be crucially dependent on Zn^2+^ were also up-regulated. Taken together, these data implicate Zur in presiding over the cellular balance between Zn^2+^ conservation and utilization. In this way, Zur regulates the induction of Zn^2+^-dependent and -independent proteins, thereby controlling the magnitude of competition for the cellular Zn^2+^ pool and ensuring essential protein functions are maintained. Collectively, this work highlights the dynamic nature of *P. aeruginosa* Zn^2+^ acquisition, and the concerted cellular response to manage cellular Zn^2+^ utilization upon Zn^2+^ depletion (summarized in Fig. 6). Overall, this study provides new insights into the mechanisms and pathways utilized by *P. aeruginosa* to survive and promulgate in environments of varying Zn^2+^ abundance, with the findings widely applicable to other prokaryotic organisms.

## Experimental Procedures

### Bacterial strains, media and growth

The wild-type *P. aeruginosa* strain used in this study was PAO1, with the Δ*znuA* deletion mutant made using PAO1 according to Choi and Schweizer^53^ using primers listed in Supplementary Table S2 online. *P. aeruginosa* was grown in a semi-synthetic cation-defined media (CDM) containing 8.45 mM Na_2_HPO_4_, 4.41 mM KH_2_PO_4_, 1.71 mM NaCl and 3.74 mM NH_4_Cl, supplemented with 0.5% yeast extract (Difco) and vitamins (0.2 μM biotin, 0.4 μM nicotinic acid, 0.24 μM pyridoxine-HCl, 0.15 μM thiamine-HCl, 66.4 μM riboflavin-HCl, and 0.63 μM calcium pathothenate) and Chelex-100 (Sigma-Aldrich) treated. CaCl_2_ and MgSO_4_ were subsequently added to 0.1 mM and 2 mM, respectively. Metal concentrations of the CDM were ascertained by inductively coupled plasma-mass spectroscopy (ICP-MS) with Zn^2+^ present at 800 nM. For routine bacterial growth, media was inoculated to OD_600_ of 0.05 using overnight culture. Cells were grown to an OD_600_ of 0.6 on an Innova 40R shaking incubator (Eppendorf) at 240 rpm, 37 °C. Whole cell metal accumulation was performed as previously described^5^ and analysed by ICP-MS on an Agilent 7500cx ICP-MS (Adelaide Microscopy, University of Adelaide).

### Expression and purification of ZnuA

Recombinant ZnuA was generated by PCR amplification of *P. aeruginosa* PAO1 *znuA* using ligation-independent cloning and primers listed in Supplementary Table S2 online, to insert the gene into a C-terminal dodecahistidine tag-containing vector, pCAMcLIC01, to generate pCAMcLIC01-ZnuA. Recombinant ZnuA expression and purification was performed essentially as described previously^54^. Recombinant ZnuA had the dodecahistidine tag removed by 1 h enzymatic digestion at a ratio of 1:25 by the histidine-tagged 3C human rhinovirus protease, at a cleavage site introduced between ZnuA and the tag. The protein was then reverse-purified on a HisTrap HP column (GE Healthcare) with the cleaved protein unable to bind to the column. Removal of the dodecahistidine tag was confirmed by the observed reduction in molecular mass on a 4–12% SDS-PAGE gel and confirmed by immunoblotting. Demetallated (apo) ZnuA was prepared by dialyzing the protein (10 ml) in a 20 kDa MWCO membrane (Pierce) against 4 L of sodium acetate buffer, pH 4.0, with 20 mM EDTA, at 25 °C. The sample was then dialyzed against 4 L of 20 mM Tris-HCl, pH 7.2, 100 mM NaCl, at 4 °C. The sample was then recovered and centrifuged at 120,000 × *g* for 10 min to remove any insoluble material. Metal content analysis was performed by ICP-MS^5^.

### Homology modelling and structural analyses

The homology model of *P. aeruginosa* ZnuA was constructed using the SwissModel webserver^55^, with ZnuA (PDB ID: 2OGW) as a template. The resulting model of ZnuA was energy-minimized in SwissPDBViewer^56^ using the inbuilt 43B1 vacuum forcefield^57^. Structure-based sequence alignment was performed with 3D-Coffee^58^ as described in Plumptre, *et al.*^36^.

### Biophysical analyses of ZnuA

Zinc loading assays were performed on 3C cleaved ZnuA (20 μM) as previously described^31^. The supernatant was then analysed by ICP-MS and the protein-to-metal ratio determined. Determination of the *K*_D_ for ZnuA with Zn^2+^ was performed by means of a competition assay using apo-ZnuA and the Zn^2+^-fluorophore Mag-Fura-2 (Life Technologies) as previously described^36^. Competition by ZnuA for Zn^2+^ binding was assessed by monitoring the increase in the fluorescence of 150 nM Mag-Fura-2-Zn^2+^ in response to increasing apo-ZnuA concentrations and analysed using log_10_[inhibitor] versus response model, with the experimentally derived *K*_D_ for Mag-Fura-2 (22.6 ± 6.4 nM, with Zn^2+^, *n* = 8) in Graphpad Prism to determine the *K*_D_ value for Zn^2+^ binding by ZnuA. The thermal shift assays were performed essentially as described previously^5^. Briefly, 10 μM of protein in 100 mM MOPS, pH 7.2, 150 mM NaCl, 5 × SYPRO Orange (Life Technologies) was incubated in the presence of 1 mM metal ion and then analysed on a Roche LC480 Real-Time Cycler (Roche). The fluorescence data were collected by excitation at 470 nm and emission at 570 nm. After subtraction of the background fluorescence from the buffer, the first derivative of the fluorescence data was determined and analysed using Graphpad Prism to determine the inflection point of the melting transition (*T*_m_). Data from at least three independent experiments were used to determine the mean *T*_*m*_ (±s.e.m.) of wild-type ZnuA.

### Zur binding site identification

The *P. aeruginosa* Zur binding motif was determined as described previously^59^. In brief, the sequences of the *P. protegens* Pf-5 Zur motif^38^ were used to generate a *P. aeruginosa* PAO1 optimized Zur binding site motif. The sequences were aligned using ClustalW2^60^ and a subsequent weight matrix was generated using HMMER 2.0 as an integral tool of UGENE^61^. Iterative refinement of the PAO1 Zur binding motif was performed based on genomic positioning, *E*-value (≤0.002) and up-regulation of the downstream gene (≥2-fold). The resulting sequences from which the Zur binding motif has been generated have been listed in Supplementary Table S1 online.

### RNA isolation

Cells were grown aerobically to OD_600_ of 0.6 as detailed above, then 5 mL culture was harvested at 7000 × *g*, for 8 min, 4 °C and lysed in Trizol reagent (Life Technologies, USA) and chloroform. Following phase separation by centrifugation, RNA was isolated from the aqueous phase using a PureLink RNA Mini Kit (Life Technologies), with a 30 min on-column DNaseI treatment with 2.7 U DNaseI. DNaseI treatment was performed on 2 μg total RNA using 50 units of recombinant RNase-free DNaseI (Roche) in a 50 μL reaction at 37 °C for 30 min, prior to inactivation of the enzyme by the addition of ethylene glycol-*bis*(2-aminoethylether)-*N*,*N*,*N′*,*N′*-tetraacetic acid (EGTA, pH 8.0) to a final concentration of 2 mM, and incubation at 65 °C for 10 min. Samples were analysed for purity and integrity using a RNA 6000 Nano Assay on a Bioanalyzer (Agilent Technologies) according to the manufacturers protocol and stored at −80 °C until required.

### qRT-PCR

For transcriptional analysis qRT-PCR was performed using a two-step method as previously described^54^. Briefly, cDNA was synthesized using random hexamers (Sigma-Aldrich) and Moloney murine leukaemia virus RNaseH minus point mutant (M-MLV, RNaseH minus) reverse transcriptase (Promega), as per the manufacturer’s protocol. Quantitative PCR was performed on a LightCycler 480 (Roche) using DyNAmo ColorFlash SYBR Green qPCR mix (ThermoFisher Scientific). Oligonucleotides used in this study were designed using Primer3 integrated within UGENE v1.11.4 (Unipro)^61^ and are listed in Supplementary Table S2 online. The constitutively expressed sigma factor gene *rpoD* (PA0576) was used as a control to normalize gene expression, with the data representing biological triplicates.

### RNA sequencing

RNA isolated from biological triplicates of wild-type PAO1 and Δ*znuA* strains was pooled and submitted to the Adelaide Microarray Centre (University of Adelaide) for sequencing. Briefly, the Epicentre Bacterial Ribozero Kit (Illumina) was used to reduce the ribosomal RNA content of the total RNA pool, followed by use of the Ultra Directional RNA kit (New England Biolabs) to generate the barcoded libraries. Prepared libraries were then sequenced using the Illumina HiSeq2500 with Version 3 SBS reagents and 2 × 100 bp paired-end chemistry. Reads were aligned to the *P. aeruginosa* PAO1 genome (GenBank accession number AE004091.2)^26^ using BOWTIE2 version 2.2.3^62^. Counts for each gene were obtained with the aid of SAMtools (v 0.1.18)^63^ and BEDtools^64^ and differential gene expression was examined using DESeq^65^; the data has been submitted to GEO (accession number GSE60177).

## Additional Information

**How to cite this article**: Pederick, V. G. *et al.* ZnuA and zinc homeostasis in *Pseudomonas aeruginosa. Sci. Rep.* **5**, 13139; doi: 10.1038/srep13139 (2015).

## Supplementary Material

Supplementary Information

## Figures and Tables

**Table 1:** Effect of first row transition metal ions on the melting temperature of apo-ZnuA.

Sample	*T*_*m*_ (°C)^a^	Δ*T*_*m*_ (°C)
apo-ZnuA	48.45 ± 1.25	—
ZnuA-Mn^2+^	49.53 ± 1.65	+1.08
ZnuA-Fe^2+^	52.58 ± 3.32	+4.14
ZnuA-Fe^3+^	44.36 ± 3.00	−4.09
ZnuA-Co^2+^	58.46 ± 1.00	+10.01
ZnuA-Ni^2+^	51.88 ± 1.66	+3.43
ZnuA-Cu^2+^	52.81 ± 0.33	+4.37
ZnuA-Zn^2+^	61.41 ± 1.12	+12.96

^a^Values shown represent the average and standard deviation from at least three independent measurements.

**Table 2:** *P. aeruginosa* gene transcription under Δ*znuA*-induced Zn^2+^ depletion.

Locus ID	Predicted functions^a^	Foldchange	Zur bindingsite (*E*-value)
PA0781	TonB-dependent receptor (ZnuD)	172.2	0.0012
PA1921	methyltransferase	15.1	0.00027
PA1922	TonB-dependent receptor	147.3	0.00027
PA1923	cobaltochelatase subunit (CobN)	122.2	
PA1924	ExbD	44.7	
PA1925	hypothetical	56.4	
PA2434	hypothetical	6.5	0.11
PA2435	P-type ATPase importer (HmtA)	5.6	
PA2437	membrane protease subunit of HflC family	14.4	
PA2438	HflC membrane protease subunit	10.6	
PA2439	membrane protease subunit of HflK family	9.0	
PA2911	TonB dependent receptor	8.1	0.00027
PA2912	nucleotide binding domain of ABC transporter	8.8	
PA2913	iron periplasmic binding protein	9.1	
PA2914	transmembrane domain of Vitamin B12 ABC permease	8.0	
PA2915	metallo β-lactamase	5.2	—
PA2916	lysine transporter (LysE)	4.2	—
PA3282	hypothetical	4.1	—
PA3283	hypothetical	5.0	
PA3284	hypothetical	5.0	
PA3600	50S ribosomal protein L36	89.2	0.0013
PA3601	50S ribosomal protein L31	109.0	
PA4063	Zn^2+^ periplasmic binding protein	45.1	0.0011
PA4064	ABC transporter nucleotide binding protein	17.2	
PA4065	lipoprotein release ABC transporter permease	22.1	
PA4066	lipoprotein	10.6	
PA4833	hemolysin III family protein	5.0	—
PA4834	putative membrane transporter	27.4	0.0014
PA4835	hypothetical	35.2	
PA4836	hypothetical	72.9	
PA4837	TonB-dependent siderophore receptor	110.0	
PA4838	hypothetical membrane protein	6.7	0.0014
PA5498	Zn^2+^ ABC transporter SBP (ZnuA)	5.2	0.0012
PA5532	cobalamin biosynthesis protein (CobW)	8.9	—
PA5534	hypothetical	57.5	0.00021
PA5535	cobalamin synthesis protein	46.1	
PA5536	Zn^2+^-independent transcription regulator (DksA2)	134.6	
PA5537	glutamine synthetase	6.7	0.00021
PA5538	N-acetylmuramoyl-L-alanine amidase (AmiA)	18.6	0.00017
PA5539	GTP cyclohydrolase (FolE2)	93.6	0.00017
PA5540	carbonate dehydratase	37.6	
PA5541	dihydroorotase (PyrC2)	37.9	
PA5542	β-lactamase	6.6	—
PA5543	hypothetical	5.4	—

^*a*^The functional prediction was determined by BLAST searches (*P* value < 10^−30^).
