# Supplementary material for: ZnuA and zinc homeostasis in Pseudomonas aeruginosa
Source: Sci Rep. 2015 Aug 20;5:13139. doi: 10.1038/srep13139 (PMC4542158; doi:10.1038/srep13139)
Supplement: Supplementary Information [file srep13139-s1.pdf]

ZnuA and zinc homeostasis in *Pseudomonas aeruginosa*

Victoria G. Pederick [1*], Bart A. Eijkelkamp [1*], Stephanie L. Begg [1], Miranda P. Ween [1], Lauren J. McAllister [1], James C. Paton [1] and Christopher A. McDevitt [1]

1. Research Centre for Infectious Diseases, School of Molecular and Biomedical Science, University of Adelaide, Adelaide, South Australia, Australia.

Corresponding author: Christopher A. McDevitt, Research Centre for Infectious Diseases, School of Molecular and Biomedical Science, University of Adelaide, Adelaide, SA, 5005, Australia.
Phone: +61 8 8313 0413; Fax: +61 8 8303 7532; Email: christopher.mcdevitt@adelaide.edu.au
* Contributed equally

Supplementary Data

Supplementary Tables S1-S2

Supplementary Figures S1-S4

SUPPLEMENTARY TABLES

Supplementary Table S1. PAO1 Zur binding site sequences

Locus tag	Putative Zur binding site
PA0781	AAGAAATAATATAACAT
PA1921/22	AAGTTATAACATAACAT
PA2911	ATGTTATACTATAACAA
PA3601	ATGTTATATAGTTACAT
PA4063	TCGTTATAACGTAACAA
PA4837/38	ATGATATACTATAACGC
PA5498/99	ACGTTATTATATTACCC
PA5536/37	ACGTTATACTATAACAA
PA5539	ACGTTATATTATAACAT

Supplementary Table S2. Oligonucleotide primers used in this study

**Name**	**Target**	**Sequence (5' → 3')**	**Source**
Gm-F	Gm^R	CGAATTAGCTTCAAAAGCGCTCTGA	(Choi & Schweizer, 2005)
Gm-R	Gm^R	CGAATTGGGGATCTTGAAGTTCCT	(Choi & Schweizer, 2005)
GW-*attB*1		GGGGACAAGTTTGTACAAAAAAGCAGGCT	(Choi & Schweizer, 2005)
GW-*attB*2		GGGGACCACTTTGTACAAGAAAGCTGGGT	(Choi & Schweizer, 2005)
PA5498-UpF-GWL	PA5498	TACAAAAAAGCAGGCTCCCTGCGTCCCTTTGCCC	This study
PA5498-UpR-GW	PA5498	TCAGAGCGCTTTTGAAGCTAATTCGGAAATTGACGAACTTGCG	This study
PA5498-DnF-GW	PA5498	AGGAACTTCAAGATCCCCAATTCGCTTGCCGGCAAGCCTTTC	This study
PA5498-DnR-GWR	PA5498	TACAAGAAAGCTGGGTGCGTCCACGCTCACGTTC	This study
qPA0576_F	*rpoD*	CGATATAGCCGCTGAGGA	(Pederick *et al.*, 2014)
qPA0576_R	*rpoD*	GAGATCGAAATCGCCAAG	(Pederick *et al.*, 2014)
qPA0781_F	*znuD*	GAACGCCGCTGTAGAGGTAT	This study
qPA0781_R	*znuD*	ACAGCCGCTTCTCCAACTAC	This study
qPA4063_F	PA4063	CTGTTAGCCCTCGTCTTGCT	This study
qPA4063_R	PA4063	AGCTCCAGTTCGAGGGTCTT	This study
qPA2435_F	*hmtA*	GATCAGCAACACCAGCAACA	This study
qPA2435_R	*hmtA*	GCATCGAGATAACCCGTACC	This study

qPA0779_F	*asrA*	GTCCAGGTAGTGGTCGAGGA	This study
qPA0779_R	*asrA*	GGAAGTCGAGGTGATGAACC	This study
qPA1927_F	*metE*	ACCCAGATCCATACGCACAT	This study
qPA1927_R	*metE*	GTTCGGGTAGTCGAACTGCT	This study
qPA3527_F	*pyrC*	GTCTTCGAGCACATCACCAC	This study
qPA3527_R	*pyrC*	TAGAAGTGCGGACGGATACC	This study
qPA3690_F	*zntA*	GAGTTGATCGAGGCGAAGTC	This study
qPA3690_R	*zntA*	GCTTCCAGTTCCACTTGCTT	This study
qPA5230_F	PA5230	GCGACGATGTTCTGGATGTA	This study
qPA5230_R	PA5230	TCGGCCTGTATACCTTCACC	This study
qPA0277_F	PA0277	GTACGCCTTCGATCTCGTTG	This study
qPA0277_R	PA0277	GTGCCGGTGAACTACAAGGT	This study
qPA5539_F	*folE2*	GGAAGACTGCATCGGACT	This study
qPA5539_R	*folE2*	CCTCGCAGAACATCAGGT	This study
qPA2911_F	PA2911	GCAACCTGATCACCCTCA	This study
qPA2911_R	PA2911	CGCTGAGCAGCTTCTTCT	This study
qPA1544_F	*anr*	CGATGACCAGCAGATGAT	This study
qPA1544_R	*anr*	GACAGGTTGACCAGGAAG	This study
PA5498LIC1F	PA5498	TGGGTGGTGGATTTCCTGAGGTCAGCGTGCTGACC	This study
PA5498LIC1R	PA5498	TTGGAAGTATAAATTTCCGAGCTTTTCCAGACAGCC	This study

SUPPLEMENTARY FIGURES

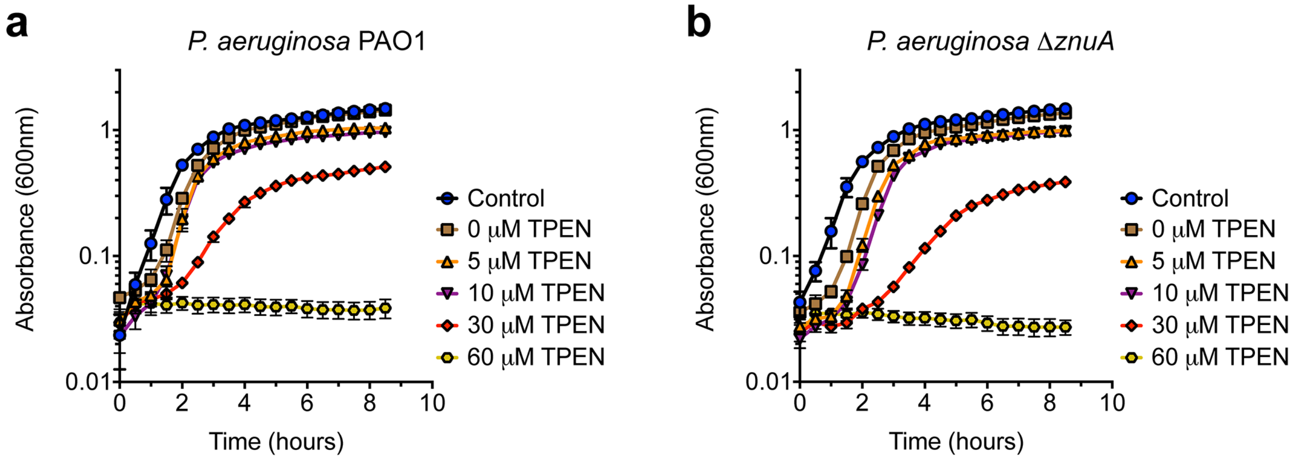

Supplementary Figure S1. The effect of Zn^{2+} limitation on *P. aeruginosa* growth. The growth phenotypes of *P. aeruginosa* PAO1 and the Δ*znuA* strain (shown in Figure 1) were compared by absorbance at 600 nm. CDM overnight seed cultures were supplemented with 0 µM TPEN (control) or 10 µM TPEN (all other treatments), prior to growth analyses in CDM supplemented with additional TPEN with concentrations as indicated. The control treatment involved no TPEN supplementation. **(a)** *P. aeruginosa* PAO1 **(b)** *P. aeruginosa* Δ*znuA*. Cultures were grown as indicated and, in all experiments, data are the mean ± s.e.m. with n ≥ 3.

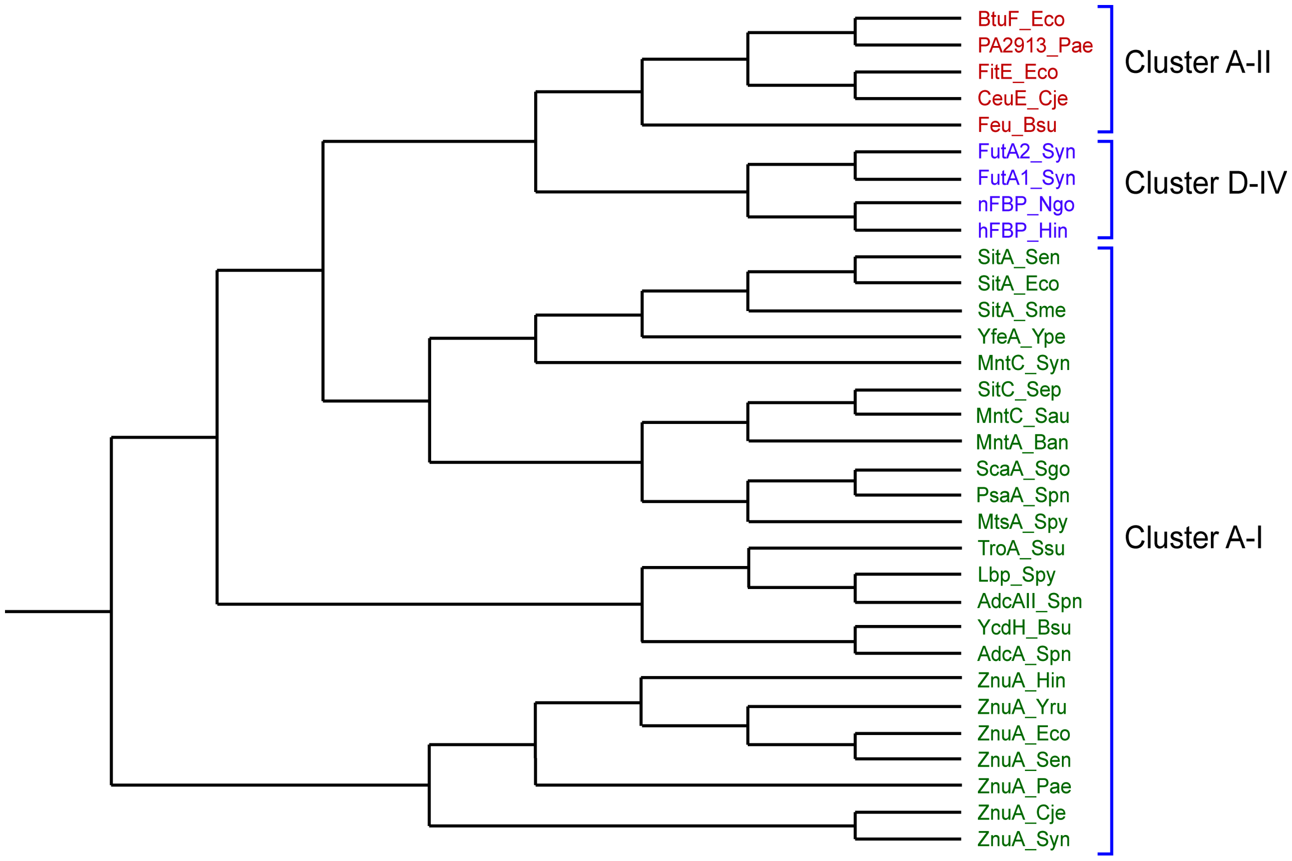

Supplementary Figure S2. Phylogenetic tree of metal binding SBPs. The phylogenetic relationship between *P. aeruginosa* PA2913 and characterized cluster A-I, A-II and D-IV iron binding SBPs was assessed. Amino acid sequences were aligned using ClustalW2 (Larkin *et al.*, 2007) and a phylogenetic tree constructed in CLC Sequence Viewer (Version 6.7.1) using the Neighbor Joining method. Proteins clustered according to their SBP classification. All cluster A SBPs feature a long α-helical region that joins the two domains of the protein. They are further subdivided into cluster A-I SBPs (green) that bind their metal ligand directly, and cluster A-II SBPs (red) that bind a chelated form of their metal ligand. The cluster D-IV SBPs (purple) are an iron-specific subset of cluster D proteins that bind iron (ferric or ferrous). BtuF_Eco (GI: 215485321, *E. coli*); PA2913_Pae (GI: 15598109, *P. aeruginosa* PAO1) FitE_Eco (GI: 190907145, *E. coli*); Ceu_Cje (GI: 112360670, *C. jejuni*); Feu_Bsu (GI: 2632430, *B. subtilis*); FutA2_Syn (GI: 170785083, *Synechocystis* PCC6803); FutA1_Syn (GI: 152149369, *Synechocystis* PCC6803); nFBP_Ngo (GI: 28948320, *Neiserria gonorrhoeae*); hFBP_Hin (GI: 157832046, *H. influenzae*);

SitA_Sen (GI: 16761638, *S. enterica*); SitA_Eco (GI: 84060846, *E. coli*); YfeA_Ype (GI: 51596671, *Y. pestis*); MntC_Syn (GI: 16330511, *Synechocystis* PCC6803); SitC_Sep (GI: 27467323, *S. epidermis*); MntC_Sau (GI: 88194402, *S. aureus*); MntA_Ban (GI: 30263122, *B. anthracis*); ScaA_Sgo (GI: 1168357, *Streptococcus gordonii*); PsaA_Spn (GI: 182684593, *S. pneumoniae*); MtsA_Spy (GI: 489075835, *S. pyrogenes*); TroA_Ssu (GI: 330832169, *S. suis*); Lbp_Spy (GI: 15675794, *S. pyrogenes*); AdcAII_Spn (GI: 15902950, *S. pneumoniae*); YcdH_Bsu (GI: 668752994, *B. subtilis*); AdcA_Spn (GI: 15904016, *S. pneumoniae*); ZnuA_Hin (GI: 491963406, *H. influenzae*); ZnuA_Yru (GI: 490857750, *Y. ruckeri*); ZnuA_Eco (GI: 635897169, *E. coli*); ZnuA_Sen (GI: 541470409, *S. enterica*); ZnuA_Pae (GI: 15600691, *P. aeruginosa* PAO1); ZnuA_Cje (GI: 504330062, *C. jejuni*), ZnuA_Syn (GI: 499174152, *Synechocystis* PCC6803).

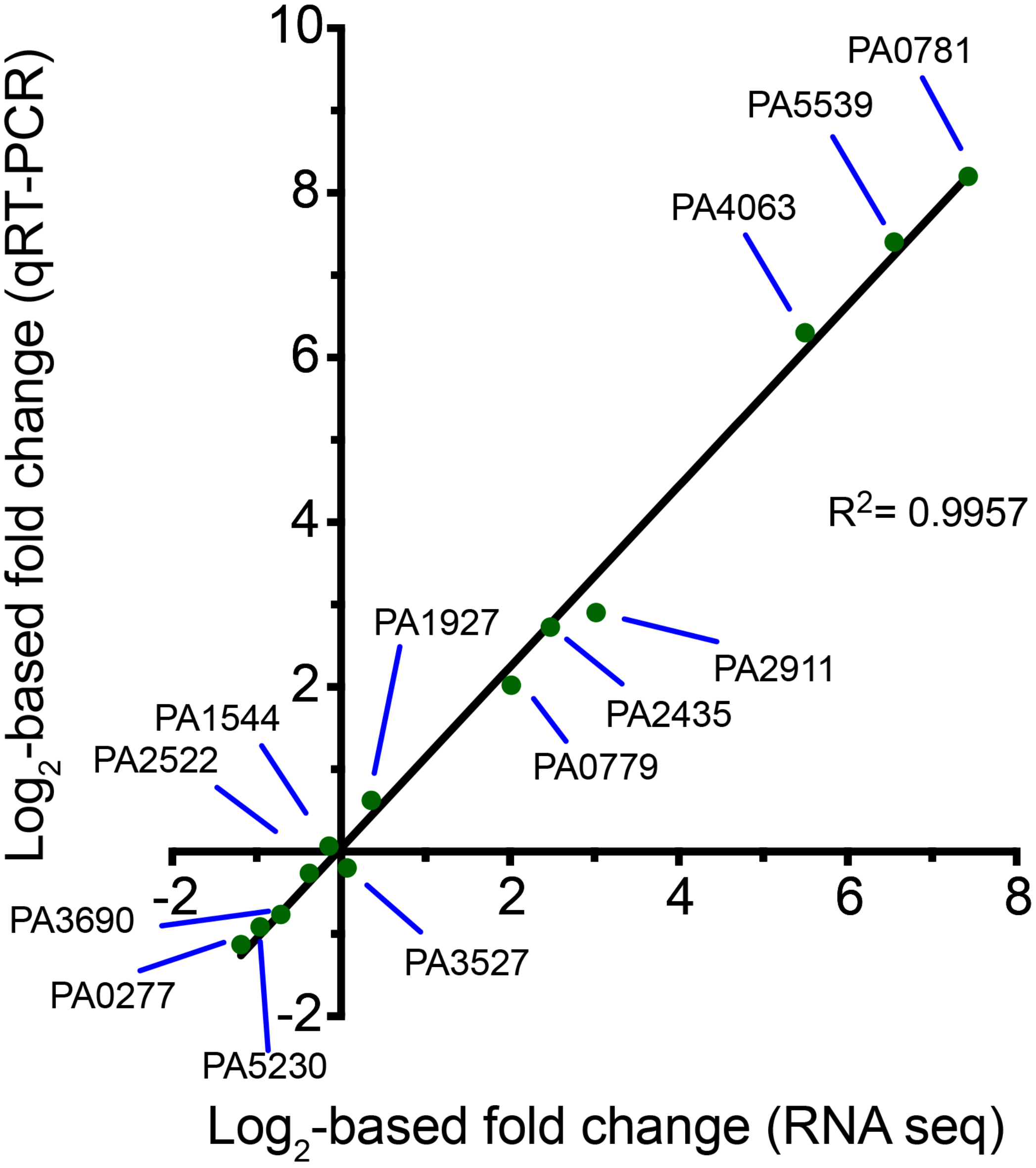

Supplementary Figure S3. Validation of RNA sequencing results. The differential expression of genes as determined by RNA sequencing was confirmed by RT-qPCR analysis of *P. aeruginosa* PAO1 and Δ*znuA* samples grown in biological triplicate. Data are expressed as the mean $\log_2$-fold change.

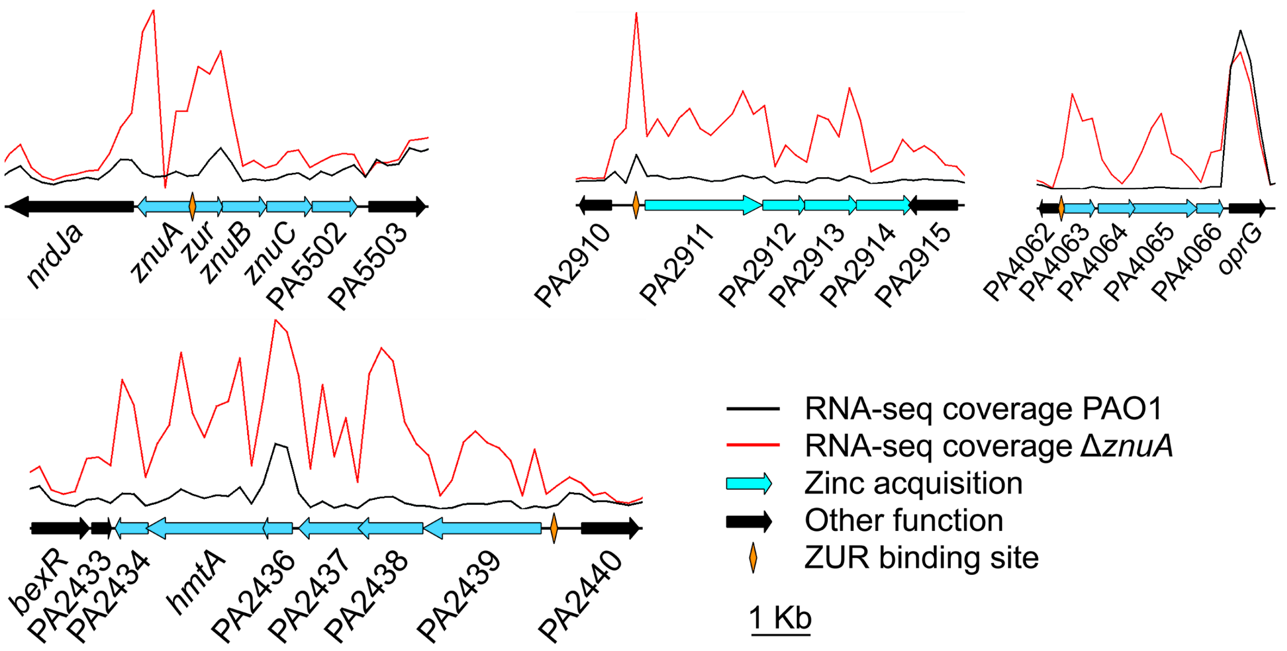

Supplementary Figure S4. mRNA levels of putative Zn^{2+} acquisition clusters in *P. aeruginosa* PAO1. Expression levels of the putative inner membrane Zn^{2+} acquisition gene clusters in the wild-type (black) and Δ*znuA* strain (red) as determined by RNA-sequencing. Genes proposed to exhibit roles in Zn^{2+} acquisition appear as blue arrows, those with alternative functions appear as black arrows and Zur binding sites are represented as orange diamonds. Genes are drawn to scale.
